# Farewell to a teacher

**DOI:** 10.15694/mep.2019.000198.1

**Published:** 2019-11-06

**Authors:** Markus Donix

**Affiliations:** 1 Department of Psychiatry

**Keywords:** teaching medicine, medical student, inspiration, development

## Abstract

This article was migrated. The article was marked as recommended.

There are frequent discussions about the challenges of teaching in medical school, often focusing on technological aspects or time constraints. The retirement of one of my mentors reminded me that being an inspirational teacher is also important for our students’ personal and professional development.

## Personal Reflection

The old lecture hall was full to the last seat. My hands felt the scars previous generations of medical students had left on the dark wooden folding tables. It was a cold October morning, and the dim light in the steep auditorium corresponded well to the world outside the large windows. Before sitting down for our very first lecture in medical school we had passed by glass cabinets with yellowed brain sections and other specimen in the hallways of the anatomical institute. When the professor entered the room, the excited chatter of more than two hundred new students was gone. He did not turn on a projector as he slowly walked to a large blackboard. With white chalk he first outlined a skull in side view, then a spinal column with all its curvatures, followed by the major limb bones in remarkably precise proportions. “The human body”, he said, and paused. “That’s how we begin”. It was so quiet that we could hear the noise of the light rain falling against the windows. In this moment I knew that it was the right decision to become a physician. I was looking forward to what would be next, and how this first anatomy lecture would blend into future clinical work.

Twenty years later I am a new academic staff member at the same university’s faculty of medicine, sitting next to the anatomy professor in one of our regular board meetings. Today I vividly remember my days as a student because we just learned that he would soon retire. He radiates confidence and composure, and he briefly smiles when the dean let us know that the faculty is already looking for a successor. I am lost in thought, as suddenly the door opens and medical students enter the conference room. They had been invited to share their perspectives on teaching issues with us. The students talk about overcrowded lecture halls, increasingly challenging curricula, and the difficulty some professors have to comprehensively present important aspects of their field in a limited amount of time. Many faculty members nod in agreement and our dean thanks the students for speaking openly. My colleagues then briefly discuss technological opportunities in modern education, the possible significance of training teachers in didactics, and how to best integrate basic science and clinical practice. However, there might be something else our students are also looking for: I am reminded again of how a few years after our first lecture in medical school, the anatomy professor invited his own former mentor for a talk at our university. We expected the emeritus, an elderly man well known for his contributions to photographic anatomy atlases, to speak about death or the preservation of human bodies. Surprisingly he did not; instead he reflected on the beginning of life, on how a single cell would mature into something beautiful and unique, and, at the same time, how that cell and its nurturing environment is all there is to differentiate from - ‘from the whole to its parts’. I am thankful for this experience, although back then I did not fully appreciate how the ideas of late 18th century romanticism might influence biological or ethical concepts. However, it was a moment that made us think about life and humanism, transcending the boundaries of a lecture hall and the time frame of a memorable speech.

As medical students we are observing and practicing the skills we need as future clinicians and scientists. We depend on the appropriate educational resources, as we will ultimately develop and differentiate into our later selves, pursuing individual strengths, interests, and careers. As teachers we should remind us that this development is always nurtured by inspiration. We will soon be with our students again - during a ward round, in a private practice or in a lecture hall. When we start teaching, we should pause for a moment, and then, the chalk is in our hands.

## Take Home Messages

• Inspirational teaching nurtures professional and personal development in medical school
• Medical school teachers should reflect on how students may perceive experienced professionals

## Notes On Contributors

Markus Donix, M.D., is a Professor of Geriatric Psychiatry and Vice Chair of the Department of Psychiatry, University Hospital Carl Gustav Carus, Technische Universität Dresden, Germany. ORCID: https://orcid.org/0000-0001-8947-0428

