# Supplementary material for: Farewell to a teacher
Source: MedEdPublish (2016). 2019 Nov 6;8:198. [Version 1] doi: 10.15694/mep.2019.000198.1 (PMC10712463; doi:10.15694/mep.2019.000198.1)
Supplement: Peer review history

# Reviewer report: Reviewer response for version 1

**DOI:** 10.21956/mep.19941.r30272

**Published:** 2019-11-25

**Authors:** P Ravi Shankar

**Affiliations:** 1 American International Medical University

This review has been migrated. The reviewer awarded 4 stars out of 5 Farewell to a beloved teacher is always a poignant moment. We had a number of farewell ceremonies at the Manipal College of Medical Sciences in Pokhara, Nepal which we attended by faculty, non teaching staff and students. My first impression of medical school (from my undergraduate days) is of the laboratories and dissection halls of the three subjects of anatomy, biochemistry and physiology. These were the preclinical subjects covered during the first eighteen months of first MBBS (the undergraduate medical course). I remember biochemistry for complicated structures and colorful reactions. My experiences of physiology are of pricking myself to get a drop of blood and of the smoked paper and the kymographs. Anatomy dissection hall was huge and had the strong smell of formalin. We first came face to face with death in the long corridors of the hall. We often get immersed in the technical details of the subject and loose our ability to wonder. How does a single fertilized human cell grow into a complex human being? It is a wonderful synchrony of complex reactions. The complete details of this process we do not know even today.

Reviewer Expertise:

NA

No decision status is available

# Reviewer report: Reviewer response for version 1

**DOI:** 10.21956/mep.19941.r30270

**Published:** 2019-11-15

**Authors:** Sateesh Babu Arja

**Affiliations:** 1 Avalon University School of Medicine

This review has been migrated. The reviewer awarded 4 stars out of 5 A very good narration. It was nostalgic and it reminded me of my first lecture in Anatomy lecture hall on November 11th, 1996. Recently all our classmates celebrated the anniversary on November 11th. I must agree with the comment made Dr. Balaji Arumgam that these events became rare after cropping of medical colleges in the private sector. I am also working in a private medical school. Still we are lucky to see that students in our medical schools do give send off/farewell for teachers who are leaving the school. We also have cultural shows, farewells for students who are leaving, celebration of festivals on campus, and community reach-out activities which nurture professional and personal development.

Reviewer Expertise:

NA

No decision status is available

# Reviewer report: Reviewer response for version 1

**DOI:** 10.21956/mep.19941.r30271

**Published:** 2019-11-07

**Authors:** BALAJI ARUMUGAM

**Affiliations:** 1 TAGORE MEDICAL COLLEGE AND HOSPITAL

This review has been migrated. The reviewer awarded 4 stars out of 5 ITS A GOOD NARRATION AND ALMOST FELT LIKE, I WAS SITTING INSIDE THE LECTURE HALL....SEND OFF, FAREWELL, THANKS GIVING PROGRAMS WERE COMMONLY DONE TO THE MOST INSPIRING TEACHERS DURING OLDEN COLLEGE DAYS. NOW A DAYS, AFTER THE CROPPING OF MEDICAL COLLEGES IN PRIVATE SECTOR, HARDLY THE TEACHERS ARE GETTING THE RECOGNITION.....BUT ANYWAY ITS GOOD opinion from you...

Reviewer Expertise:

NA

No decision status is available
